# Preparation of VZrHfNbTa High-Entropy Alloy-Based High-Temperature Oxidation-Resistant Coating and Its Bonding Mechanism

**DOI:** 10.3390/ma16175976

**Published:** 2023-08-31

**Authors:** Mengjun Hu, Rui Tan, Xiaojuan Jiang, Mengyao Dong, Junyu Chen, Meilong Hu, Yu Yang

**Affiliations:** 1 School of Mechanical Engineering and Automation, Chongqing Industry Polytechnic College, Chongqing 401120, China; humj@cqipc.edu.cn (M.H.); jiangxj@cqipc.edu.cn (X.J.); dongmy@cqipc.edu.cn (M.D.); 2 School of Materials Science and Engineering, Chongqing University, Chongqing 400044, China; 18269785642@163.com (R.T.); cjy8535@outlook.com (J.C.); 3 Comprehensive Testing and Analyzing Center, North China University of Science and Technology, Tangshan 063210, China

**Keywords:** high-entropy alloys, oxidation, surface, principal component analysis, mechanical properties, composites

## Abstract

Ultra-high Temperature Oxidation-Resistant Alloys (UTORAs) have received a lot of attention due to the increased research demand for deep space exploration around the world. However, UTORAs have the disadvantages of easy oxidation and chalking. So, in this study, a UTORAs is prepared by hot-press sintering on VZrHfNbTa (HEA: High Entropy Alloys can generally be defined as more than five elements by the equal atomic ratio or close to the equal atomic ratio alloying, the mixing entropy is higher than the melting entropy of the alloy, generally forming a high entropy solid solution phase of a class of alloys.) a substrate coated with hafnium. The bonding mechanism, resistance to high-temperature oxidation, and hardness of the sample tests are carried out. The results show that zirconium in the matrix will diffuse into the hafnium coating during the high-temperature sintering process and form the HfZr alloy transition layer, the coating thickness of the composite is about 120 μm, and the diffusion distance of zirconium in the hafnium coating is about 60 μm, this transition layer chemically combines the hafnium coating and the HEA substrate into a monolithic alloy composite. The results of high-temperature oxidation experiments show that the oxidation degree of the hafnium-coated VZrHfNbTa composite material is significantly lower than that of the VZrHfNbTa HEA after oxidation in air at 1600 °C for 5 h. The weight gain of the coated sample after oxidation is 56.56 mg/cm^2^, which is only 57.7% compared to the weight gain of the uncoated sample (98.09 mg/cm^2^ for uncoated), and the surface of the uncoated HEA shows obvious dents, oxidation, and pulverization occurred on the surface and interior of the sample. In contrast, the coated composite alloy sample mainly undergoes surface oxidation sintering to form a dense HfO_2_ protective layer, and the internal oxidation of the hafnium-coated VZrHfNbTa composite alloy is significantly lower than that of the uncoated VZrHfNbTa HEA.

## 1. Introduction

With the continuous exploration of extreme environments such as deep space, deep sea, and outer space, there is an increasing requirement for functional materials capable of withstanding ultra-high and ultra-low temperatures. High-temperature alloys are the main materials serving as core components in frontier technologies such as aerospace and nuclear industries, such as engines, gas turbines, and high-temperature structural parts. These alloys require high strength, high-temperature fatigue resistance, oxidation resistance, and thermal corrosion capability. However, high-temperature and oxidation have become the primary problems that limit the further application of high-temperature alloys in extreme environments.

Comprehensive consideration of existing high-temperature oxidation-resistant materials, the main high-temperature oxidation-resistant alloys used in engineering are iron, cobalt, and nickel-based alloys. The cost of iron-based high-temperature alloys is relatively lower, but poorer high-temperature stability. FeAl intermetallic compounds [1] have better temperature resistance and excellent mechanical properties than ceramic materials, but they are limited by their inherent brittleness. Cobalt-based high-temperature alloys are strengthened mainly by the precipitation of carbides and a solid solution of refractory elements [2,3]. There are also imbalances in the performance of both the results of the two strengthening methods and the results of the improved experiments [2,4,5,6]. Their high-temperature stability and mechanical properties are much lower than those of nickel-based high-temperature alloys. Nickel-based high-temperature alloys have excellent comprehensive properties, and they are widely used high-temperature structural materials at present [7]. Therefore, niobium-based alloys have received attention due to the lower liquid-phase line temperature and the limitation of their melting point, but their limit service temperature [8] can no longer meet the temperature-bearing capacity required for advanced aero-engines [9]. Although niobium-based alloys have ideal high-temperature mechanical properties [10,11], they suffer from serious oxidation problems at high temperatures [12,13], for example, niobium-silicon-based alloys. The alloys currently used in industry have problems in the high-temperature oxidation environment such as pulverization and mechanical property imbalance. It has been reported [14,15,16] that an alloy material resistant to high-temperature oxidation has been developed by scholars internationally and it can operate at 1800–2000 °C, but the material has not been found domestically. Therefore, this study aims to establish an experimental foundation for subsequent research.

In recent years, with a lot of research on High-Entropy Alloys(HEAs), refractory HEAs have received attention due to their excellent properties. The emerging RHEA in recent years has good mechanical properties below 1000 °C [17], but its strength decreases greatly at ultra-high temperatures. LDHEA is difficult to prepare, uneven in structure and composition to varying degrees, and difficult to apply to ultra-high temperatures [18]. 3D transition metal HEAs have a wide range of applications [19], a popular type in the field of high entropy alloys. However, in the ultra-high temperature environment, it still faces the same problem as the above high-temperature alloy. For instance, alloys such as NbMoTaW and NbMoTaWV can operate at 1600 °C space although they show a decrease in performance [20], which surpasses the working temperature of existing alloys. HEAs offer the potential to sustain materials’ function properly in ultra-high temperature environments. Among all the publicly reported HEA systems, the constituent elements are mainly refractory metal elements such as titanium, zirconium, niobium, molybdenum, hafnium, tantalum, wolfram, vanadium, etc., which have higher melting points and strength, and excellent properties at higher temperatures, making them one of the most promising high-temperature structural metals [21,22,23,24]. Currently, the most effective measure to prevent HEA materials’ oxidative failure is to prepare antioxidant coatings on the material surface [25]. In this paper, the preparation of HEA-based hafnium coatings and related properties are carried out by using a hot-pressure sintering process to provide some research basis for the study of high-temperature resistant composites.

## 2. Experiment

**Figure 1:**
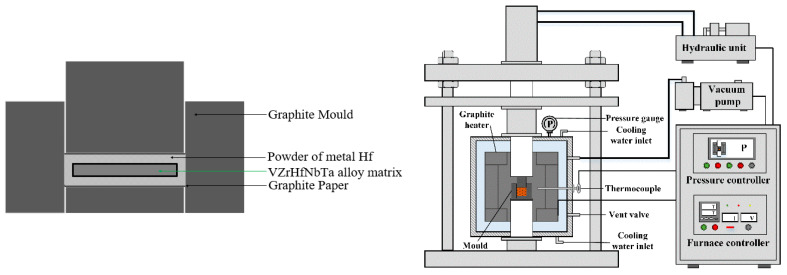
Schematic diagram of coating preparation by a hot-press sintering process.

In this study, VZrHfNbTa HEA-based surface metal hafnium coating material is prepared by the hot-press sintering process, and the preparation method can be described as the “embedding hot-press method”, as shown in Figure 1. Prepared VZrHfNbTa HEA powder (synthesized by the electro-deoxidation process) is first compressed into a cylindrical shape using a metal-chromium steel mold with a diameter of 8 mm, applying a pressure of 4 MPa. Prepared metal hafnium powder (synthesized by electro-deoxidation) is uniformly laid flat on the bottom of a cylindrical graphite mold with a diameter of 10 mm, and the powder is separated from the mold by carbon paper. Then the pressed block of VZrHfNbTa HEA is laid flat on the metal hafnium powder. Finally, press into the indenter, the mold is hot pressed and sinter at high temperature in a hot press sintering furnace. In the hot pressing sintering process, the set temperature is 1823 K with a 10 K/min heating rate, the pressure is 30 MPa, and the holding pressure is kept for 1 h at 1823 K. After finishing, the sample is cooled to normal temperature by cooling in the furnace. Cooling from 1823 K to 333 K takes 120 min, and the cooling rate is 12.4 K/min.

## 3. Results and Discussion

**Figure 2:**
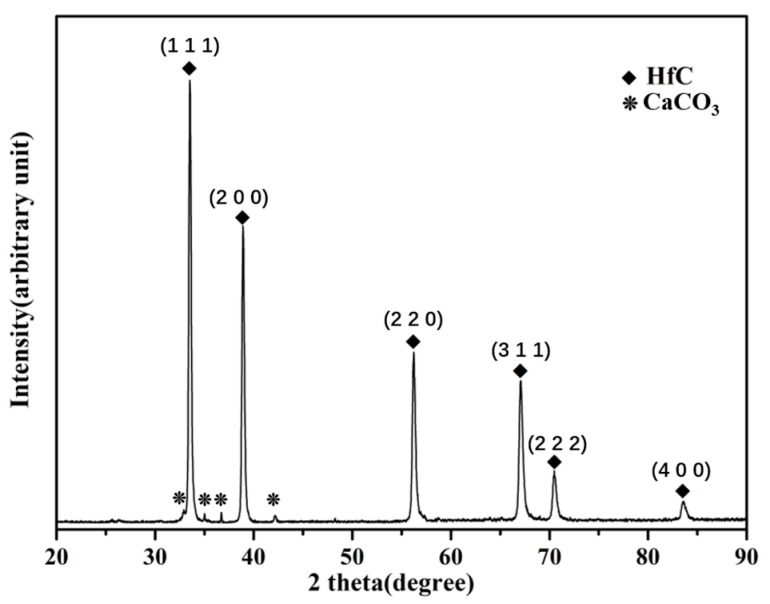
XRD pattern of the hafnium-coated VZrHfNbTa.

Figure 2 shows the X-ray Diffraction (XRD) spectrum of the surface of the Hf-coated sample prepared by hot-press sintering. The XRD analysis software we used is version 6.0 of Jade. This suggests that the main phase of the surface coating is HfC, and it is easy for hafnium to react with carbon to form carbide in the carbon environment, which can increase the hardness of the coating. The carbon environment mentioned here is provided by the graphite paper that separates the sample powder and the graphite mold. Based on the thermodynamic calculations of the equilibrium module in the software Factsage 8.1, the value of the Gibbs free energy for the reaction of hafnium with carbon to form an HfC solid at 1600 °C is −57.6 KJ/mol, which means that the reaction can proceed spontaneously at 1600 °C.

**Figure 3:**
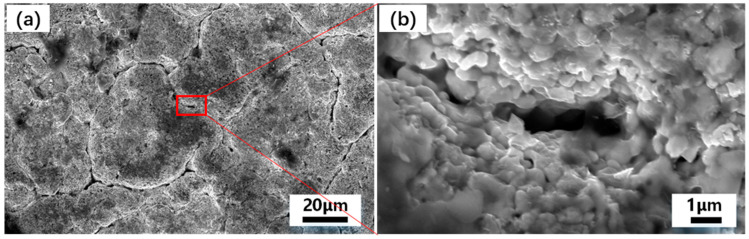
SEM images of the coating of the sample prepared by hot-press sintering process: (**a**,**b**) SEM images of the coating of the sample prepared by hot-press sintering process: (**a**) low-magnification view of Hf coating at 20 μm; (**b**) high-magnification view of Hf coating at 1 μm.

Figure 3 shows the surface Scanning Electronic Microscopy (SEM) photographs of the Hf-coated samples prepared by hot-press sintering. For the selected area in Figure 3a, we further enlarge the image. The presence of pits in the edge areas in Figure 3a is due to the fact that individual areas of hafnium powder are not covered enough to form a coating. As seen in Figure 3a, there are cracks on the surface of the coating. This is due to the sintering volume shrinkage of the high melting point HfC in the coating at 1823 K. Further magnification of the area of the cracks reveals the internal sintering, as shown in Figure 3b. The metal hafnium powder particles forming the coating are bonded to each other, and the diameter of small particles is less than 1 μm. The width of the cracks is less than 1 μm, which ensures the eminent density and strength of the coating.

**Figure 4:**
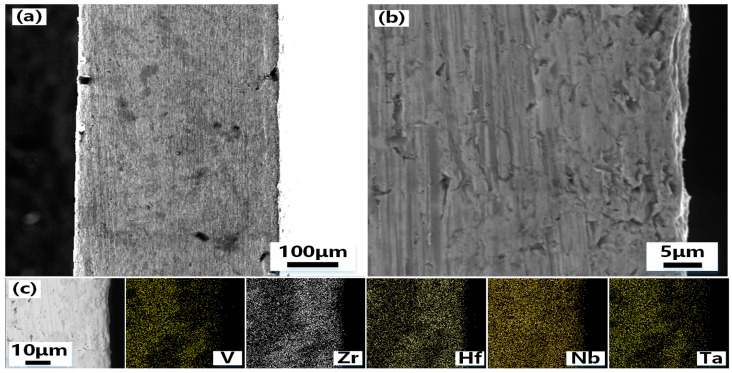
SEM images and EDX analysis of the cross-section of the VZrHfNbTa HEA: (**a**) low-magnification view of the cross-section of VZrHfNbTa high entropy alloy; (**b**) high-magnification view of the cross-section of the edge side of the VZrHfNbTa high entropy alloy; (**c**) EDX of V, Zr, Hf, Nb and Ta elements in cross sections of bulk high-entropy alloys.

**Figure 5:**
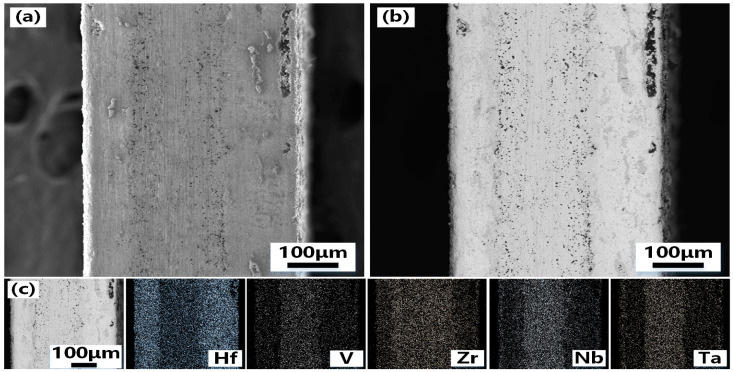
(**a**) SEM, (**b**) BSE images, and (**c**) EDX analysis of the coating sample cross-section: (**a**) SEM of sample cross-section of alloy substrate with Hf coating; (**b**) BSE of sample cross-section of alloy substrate with Hf coating; (**c**) EDX of V, Zr, Hf, Nb, and Ta elements in the cross-section of high entropy alloy possessing Hf coating.

To clarify the composite mechanism between the coating prepared by the hot-press sintering process and the matrix VZrHfNbTa HEA. The cross-section of the samples is characterized and analyzed. Figure 4 shows the SEM photos of the cross-section of VZrHfNbTa alloy and the energy spectrum analysis. Figure 4a,b show the cross-section and the side of the cross-section near the edge of the VZrHfNbTa alloy prepared by hot-press sintering, respectively, the alloy layer exhibits a dense surface, indicating an excellent hot-press sintering effect. The cross-sectional energy spectrum surface scan analysis shows that the elements of zirconium, hafnium, and niobium are uniformly distributed, while vanadium and tantalum are less concentrated near the surface. Figure 5 shows the SEM, Back-Scaterred Electron (BSE), and energy spectrum analysis of the cross-section of the Hf-coated samples prepared by hot-press sintering. As shown in Figure 5a,b, the center of the alloy cross-section has a clear boundary with the edge, and the thickness of the central part is about 150 μm. From the energy spectral surface scan of Figure 4c, a layer formed from metal hafnium with a thickness of approximately 120 μm is formed on the surface of the substrate VZrHfNbTa alloy. The energy spectrum analysis shows that vanadium, niobium, and tantalum elements have clear boundaries with the coating. Therefore, no diffusion of elements is observed during the hot pressing sintering process, and the hafnium in the coating has a distinct boundary due to its different concentration compared to the substrate alloy. In contrast, the boundary between zirconium and the coating in the cross-section is very blurred, which indicates a significant diffusion and migration of zirconium of the substrate alloy to the coating occurred during the hot-press sintering process. The generation of blurring represents the diffusion of zirconium and the possibility of forming zirconium compounds. HfZr plays an important role in the development of deep space. Therefore, this blurring is of great significance.

**Figure 6:**
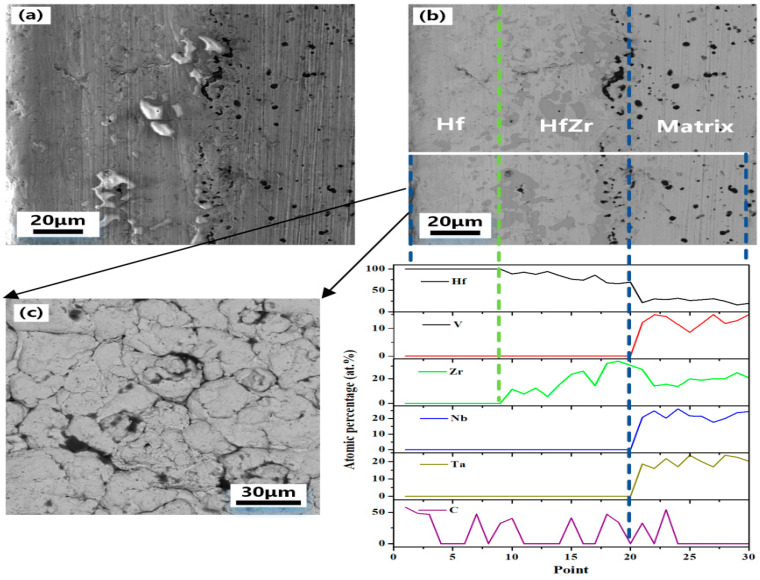
(**a**) SEM image of a cross-section of alloy sample with Hf coating. (**b**) BSE image and line scan image of EDS of a cross-section of alloy sample with Hf coating. (**c**) SEM image of Hf coating surface.

To further clarify the distribution of elements at the interface between the coating and the substrate, energy spectral line scan analysis is applied to the cross-section of the coated sample. Figure 6 shows the SEM, BSE, and energy spectrum line scan analysis of a half cross-section of the hafnium-coated sample prepared by hot-press sintering. As shown in Figure 6b, the phase difference between the substrate and the coating can be observed, but the boundary is not obvious. The white line in Figure 6b is the energy spectrum scan line. According to the content data of the line scan elements, the cross-section is divided into three parts: alloy matrix, HfZr coating, and hafnium coating from inside to outside. Based on the distribution of the zirconium element, it can be concluded that the diffusive distance of the zirconium element is about 60 μm when hot-press sinter at 1823 K for 1 h. The migration of zirconium element at the interface between the coating and the substrate helps to improve the binding force between the coating and the substrate, to effectively improve the protection of the coating on the substrate. This shows that the composite mechanism is affected by elements and diffusion distance. When there are elements in the matrix material that are easy to diffuse and can be alloyed with the coating material, a gradient composite layer can be formed between the coating and the substrate, thereby improving the binding force between the coating and the substrate. The distribution of carbon element, indicate that hafnium on the coating surface and carbon paper formed hafnium coating during the hot-press sintering process, but the thickness of HfC coating is less than 10 μm due to the limited diffusive distance of carbon element. The carbon at the interface between the substrate and the coating mainly originates from the mixture of metal hafnium prepared by electro-deoxygenation and HfC powder in the coating, and the diffusive distance is similar to the thickness of the surface HfC. The cathode carbon deposition of the electrode-oxidation process is caused by the side reaction of soluble CO^3−^ and calcium ions, which can be described by the following reactions:

(1) $$\mathrm{Anode}\;\mathrm{reaction}:\;C+2O^{2-}-4e^{-}={\mathrm{CO}}_{2}$$

(2) $$\mathrm{CaO}+{\mathrm{CO}}_{2}={\mathrm{CaCO}}_{3}$$

(3) $$\mathrm{Cathode}\;\mathrm{reaction}:{\;\mathrm{CO}}_{3}^{2-}+4e^{-}=C+3O^{2-}$$

The deposited carbon forms HfC with the metal Hf prepared by cathode deoxidation and the HfC coating is formed in the process of hot-pressing sintering to prepare the coating.

The migration of zirconium elements at the interface between the coating and the substrate helps to improve the bondability between the coating and the substrate, thus effectively improving the protection of the coating against the substrate. Figure 6b illustrates that gradient HfZr alloy is formed in the coating, which enhances the adhesion between the coating and the substrate and solves the problem of peeling that commonly occurrs in traditional alloy coating. In the VZrHfNbTa system, zirconium and hafnium belong to the same main group with similar physical and chemical properties, Although the atomic numbers of zirconium and hafnium are very different, the ionic radius is almost the same, are positive quadrivalent in the compound, and hafnium can easily replace zirconium in any compound of zirconium, which is why hafnium and zirconium always symbiosis together, and Table 1 shows the basic parameters of the alloying elements. The closer the atomic radii between the alloying elements in HEAs, the more similar type of crystal structure between the group elements, the smaller the electronegativity difference, the easier it is for two infinite intercalations, then the more favorable to the formation of solid solution. According to the Miedema model [26], it is known that the enthalpy of mixing of Hf-Zr is 0, therefore, the two elements tend to form a stable solid solution. Only the zirconium and hafnium undergo the BCC→HCP isomeric transition at low temperatures as well. After the HCP solid solution is formed, there will be serious lattice distortion. Lattice distortion causes lattice parameters to change, then the gap between particles corresponding to the larger lattice parameters becomes larger, and it is easy to become a diffusion channel, which is conducive to ion migration and diffusion coefficient. Lattice distortion here promotes zirconium diffusion. Lattice distortion causes the position of the atoms in the crystal to change, and in the region of lattice distortion, the lattice constant changes, which affects the jump frequency and energy of the atoms, and thus the rate of diffusion of the zirconium element.

**Figure 7:**
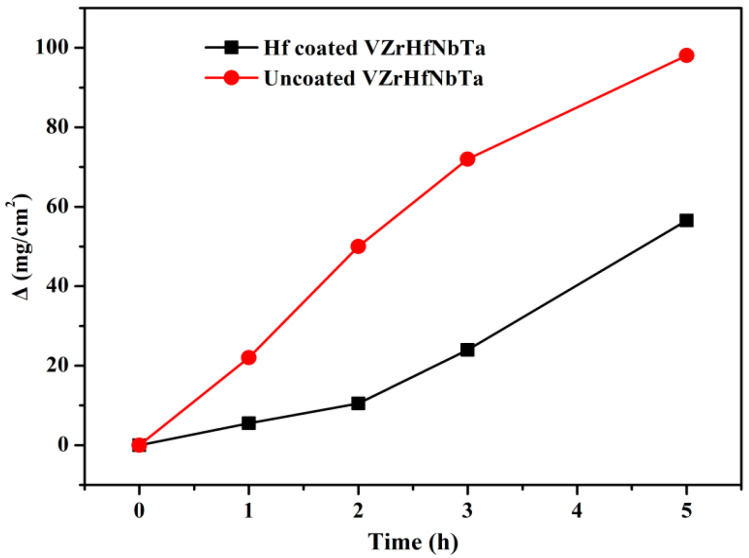
Weight change curves obtained for uncoated and coated VZrHfNbTa specimens during isothermal oxidation at 1873 K in air.

**Figure 8:**
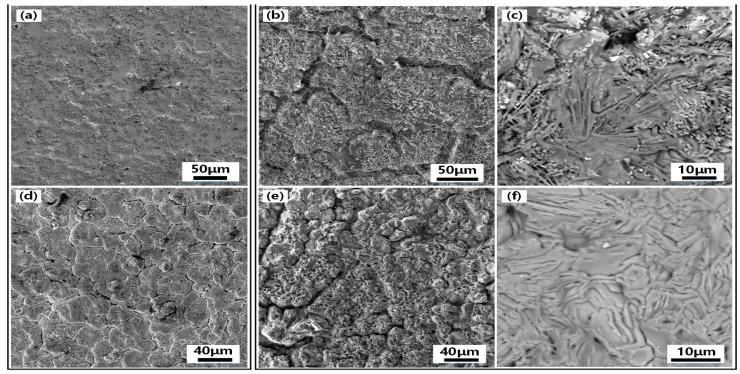
SEM and BSE images of uncoated specimen surface before and after oxidation and SEM and BSE images of Hf coated specimen surface before and after oxidation (**a**) SEM image of the surface of uncoated alloy specimen before oxidation, (**b**) SEM image of the surface of uncoated alloy specimen after 5 h of oxidation, (**c**) high-magnification BSE image of the surface of uncoated alloy specimen after 5 h of oxidation; (**d**) SEM image of the surface of the alloy specimen with Hf coating before oxidation, (**e**) SEM image of the surface of the alloy specimen with Hf coating after 5 h of oxidation, (**f**) High magnification BSE image of the surface of the alloy specimen with Hf coating after 5 h of oxidation.

In a certain period, Figure 7 shows the weight change curves of the specimens with and without coating during isothermal oxidation in air at a temperature of 1873 K. With the extension of oxidation time, the rate of the sample with Hf coating weight gain increased, while the rate of the sample without Hf coating weight gain slows down. The hafnium coating pattern forms a dense HfO_2_ oxide layer after oxidation, which can protect against the deep oxidation inside the pattern. The presence or absence of hafnium on the surface of the sample, and the formation of an HfO_2_ film during the oxidation process lead to different weight gain rates for uncoated and coated samples. Two samples may reach an equal weight after a long enough oxidation time, but the coated sample still has better high-temperature oxidation resistance and mechanical properties. It can be seen that the weight gain per unit area of the Hf-coated alloy is significantly smaller than that of the uncoated VZrHfNbTa alloy. Figure 8 shows SEM comparisons of the surface of specimens with and without hafnium coating before and after oxidation. The surface SEM of the specimen before oxidation shows that the uncoated VZrHfNbTa alloy in Figure 8a has a dense surface, in contrast, the Hf-coated specimen in Figure 8d has chaps due to the sintering volume shrinkage. This is because the melting point of Hf coating is higher than the hot pressing sintering temperature, and the Hf powder particles can be fully sintered and densified during the hot pressing sintering process, so cracks appear between particles that are not completely sintered. After oxidation for 5 h, the surface of the uncoated VZrHfNbTa alloy is shown in Figure 8b, it illustrates obvious dents and surface pulverization, and in Figure 8e, it can be observed that the Hf-coated specimen underwent obvious oxidation sintering, characterized by the disappearance of surface pulverization and appearance of ablation marks. The BSE photograph of the specimen surface at high magnification after oxidation is shown in Figure 8c, the surface erosion of the uncoated VZrHfNbTa alloy is obvious and different phases are produced. The energy spectrum analysis reveals the presence of a large amount of oxygen on the surface of the alloy. This indicates that severe oxidation occurred in the VZrHfNbTa alloy, and the different oxidation degrees of the different elements led to the pulverization of the alloy surface. The surface of the Hf-coated specimen in Figure 8f is dense and there is no other phase generation. The energy spectrum analysis shows that a dense HfO_2_ protective layer is formed on the surface of the Hf-coated specimen after oxidation, it prevents the oxygen in the air from contacting the substrate inside the coating, improving the oxidation resistance of the substrate. Therefore, hafnium and a minor amount of HfC in the coating are oxidized to form HfO_2_ and induce a sintering reaction on the coating surface.

**Figure 9:**
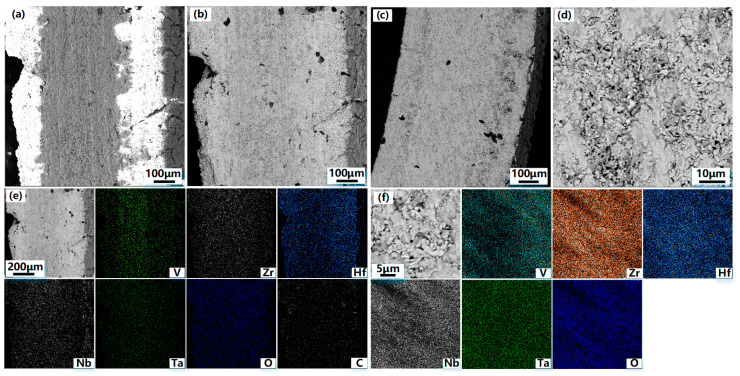
SEM, BSE images, and EDX analyses of the coated specimen cross-section after oxidation (**a**) SEM image of coated specimen sections after oxidation; (**b**) BSE image of coated specimen sections after oxidation; (**e**) EDX images of individual elements of coated specimen sections after oxidation; (**c**,**d**,**f**) SEM, BSE images and EDX analyses of the uncoated specimen cross-section after oxidation; (**c**) SEM image of uncoated specimen sections after oxidation; (**d**) high-magnification BSE image of uncoated specimen sections after oxidation; (**f**) EDX images of individual elements of uncoated specimen sections after oxidation.

**Figure 10:**
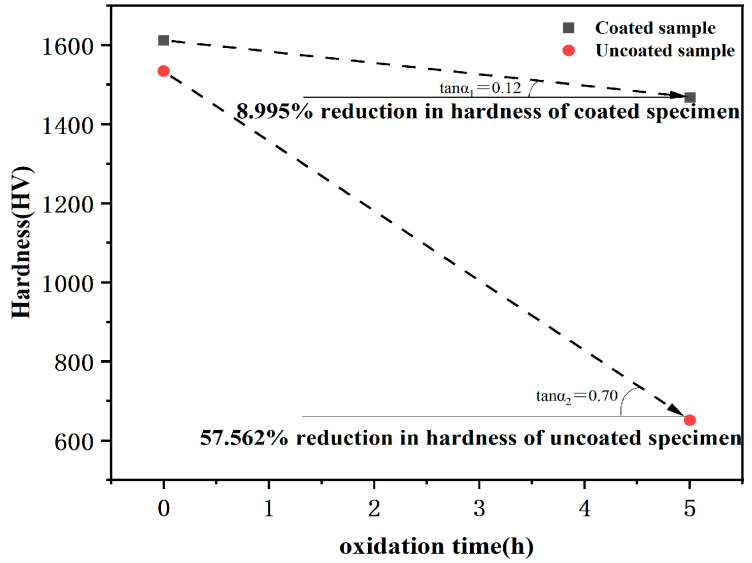
Comparison curves of surface hardness changes of coated and uncoated samples during air isothermal oxidation at 1873 K.

The specimen sections are analyzed to determine the condition of oxidation inside the two specimens. Figure 9 shows the SEM and BSE images of the coated and uncoated specimens’ cross-sections after oxidation and the respective energy spectrum analyses. From the BSE photograph of the coated specimen cross-section in Figure 9b, the phase difference between the substrate and the coating cannot be distinguished after oxidation for 5 h. The energy spectrum analyses in Figure 9e shows that most elements diffuse significantly and cannot be distinguished from the boundary of the coating, except vanadium, which has a clear boundary with the coating. This is due to the significant diffusion and migration of the elements within the specimen at the high temperature of 1873 K. However, the poor compatibility of vanadium with other alloying elements results in the slow diffusion and migration of vanadium. The distribution of oxygen elements shows that a certain degree of oxidation occurred in the overall cross-section of the coated specimen. From the high magnification BSE image of the cross-section of the uncoated specimen in Figure 9d, it can be seen that the interior of the alloy shows oxidation and pulverization as the same as the alloy surface. The energy spectrum analyses in Figure 9f show that the internal alloy experiences severe oxidation additionally, the uncoated specimen has a notably higher oxygen content compared to the coated specimen. The surface hardness of the specimens is further compared before and after oxidation, and Figure 10 shows the comparison of the surface hardness of specimens with and without hafnium coating before and after oxidation. The results indicate the surface hardness of the coated specimen decreased by 145 HV, which decreases from 1612 HV before oxidation to 1467 HV after oxidation, with a reduction of 8.995%; the surface hardness of the uncoated VZrHfNbTa HEA decreased by 883 HV, which decreases from 1534 HV before oxidation to 651 HV after oxidation, with a reduction of 57.562%, and the specimen experiences significant deformation. This can be attributed to the oxidation and pulverization of the VZrHfNbTa HEA. The hardness of the coated specimen changes less, possibly due to the HfC and hafnium in the coating forming a dense HfO_2_ protective layer after oxidizing and sintering.

## 4. Conclusions

High-temperature oxidation-resistant VZrHfNbTa refractory HEA-based Hf-coated composites are prepared by the combined process. It has a coating thickness of 120 μm. Vanadium, niobium, tantalum and hafnium elements in the base alloy have obvious boundaries with the hafnium coating, and the zirconium element undergoes diffusion and migration in the coating with a diffusive distance of about 60 μm. High-temperature oxidation tests show that the weight gain per unit area of the coated sample is much less than that of the uncoated sample. Dents and pulverization on the surface of the alloy after oxidation of the uncoated specimen, oxidation, and sintering on the surface of the coated specimen result in the formation of a dense HfO_2_ protective layer. The coating can effectively improve the oxidation resistance of the material in extreme environments and prevent the surface from chalking, thus extending the material’s service life and broadening the application. After oxidation, most of the elements in the coated specimen diffuse significantly and the boundaries with the coating are indistinguishable, except for the vanadium element in the coated specimen, which does not diffuse significantly and has a clear boundary with the coating. Oxidation and pulverization are observed inside the uncoated specimen, and the oxidation inside the alloy is more severe than that of the alloy matrix inside the coated specimen. The surface hardness of the uncoated specimen decreases significantly after oxidation, while the coated specimen changes less.

## Figures and Tables

**Table 1:** Basic parameters of alloying elements.

Elements	V	Zr	Hf	Nb	Ta
atomic mass (g/mol)	50.94	91.22	178.49	92.91	180.9
Atomic radius (nm)	0.132	0.158	0.159	0.143	0.147
Density (g/cm^3^)	5.80	6.49	13.1	8.55	16.6
Melting point (K)	2175	2125	2500	2740	3287
Boiling point (K)	3682	4682	4876	5017	5731
Crystal structure (Low Temperature)	BCC	HCP	HCP	BCC	BCC
Crystal structure (High temperature)	BCC	BCC	BCC	BCC	BCC
Crystal structure transition temperature (K)	—	1135	2033	—	—
Lattice constants (nm)	0.304	0.361	—	0.330	0.330
Electronegativity	1.63	1.33	1.3	1.6	1.5

## Data Availability

Not applicable.
